# Association of Gut Microbiome Dysbiosis with Neurodegeneration: Can Gut Microbe-Modifying Diet Prevent or Alleviate the Symptoms of Neurodegenerative Diseases?

**DOI:** 10.3390/life11070698

**Published:** 2021-07-15

**Authors:** Li Yang Tan, Xin Yi Yeo, Han-Gyu Bae, Delia Pei Shan Lee, Roger C. Ho, Jung Eun Kim, Dong-Gyu Jo, Sangyong Jung

**Affiliations:** 1 Institute of Molecular and Cell Biology (IMCB), Agency for Science, Technology and Research (A*STAR), Singapore 138667, Singapore; e0030861@u.nus.edu (L.Y.T.); xinyi.yeo12@sps.nus.edu.sg (X.Y.Y.); 2 Department of Psychological Medicine, Yong Loo Lin School of Medicine, National University of Singapore, Singapore 119228, Singapore; pcmrhcm@nus.edu.sg; 3 School of Pharmacy, Sungkyunkwan University, Suwon 16419, Korea; baehg159@gmail.com; 4 Department of Food Science and Technology, Faculty of Science, National University of Singapore, Singapore 117542, Singapore; lee.delia@u.nus.edu; 5 Institute for Health Innovation & Technology (iHealthtech), National University of Singapore, Singapore 117599, Singapore; 6 Department of Physiology, Yong Loo Lin School of Medicine, National University of Singapore, Singapore 117593, Singapore

**Keywords:** gut microbiome, gut–brain axis, neurodegenerative disease, prebiotics foods

## Abstract

The central nervous system was classically perceived as anatomically and functionally independent from the other visceral organs. But in recent decades, compelling evidence has led the scientific community to place a greater emphasis on the role of gut microbes on the brain. Pathological observations and early gastrointestinal symptoms highlighted that gut dysbiosis likely precedes the onset of cognitive deficits in Alzheimer’s disease (AD) and Parkinson’s disease (PD) patients. The delicate balance in the number and functions of pathogenic microbes and alternative probiotic populations is critical in the modulation of systemic inflammation and neuronal health. However, there is limited success in restoring healthy microbial biodiversity in AD and PD patients with general probiotics interventions and fecal microbial therapies. Fortunately, the gut microflora is susceptible to long-term extrinsic influences such as lifestyle and dietary choices, providing opportunities for treatment through comparatively individual-specific control of human behavior. In this review, we examine the impact of restrictive diets on the gut microbiome populations associated with AD and PD. The overall evidence presented supports that gut dysbiosis is a plausible prelude to disease onset, and early dietary interventions are likely beneficial for the prevention and treatment of progressive neurodegenerative diseases.

## 1. Introduction

The human gut is home to trillions of symbiotic microbes that affect digestive-related functions, modulate the structural integrity of the gut mucosal barrier, and serve immunomodulation purposes for protection against pathogenic infections [1]. Disruption of the various gut microbes, multiple host-to-microbe, and interspecies interactions are widely purported to lead to complex physiological outcomes in the gut and within the human system. Age and health-dependent microbial competition can further perturb spatiotemporal biodiversity and drive the development of unique microbiome profiles in closely similar individuals [2]. The maintenance of a healthy gut microbiome is therefore important, as dysregulation of the microbiome populations is widely implicated across many acute and chronic diseases. Prolonged use of antibiotics is one prominent example: long-term antibiotics treatments can lead to reduced species diversity and alterations in general metabolic activity, and the acquisition of antibiotic-resistance genes [3]. Some of these outcomes can be malignant, as seen in the emergence of antibiotic-resistant recurrent *Clostridium difficile* infections [4]. Alterations in microbial metabolic activity have also been associated with other systemic, immunological, and neurological complications such as bowel mobility disorders, Type 2 diabetes, and non-alcoholic fatty liver disease.

**Figure 1:**
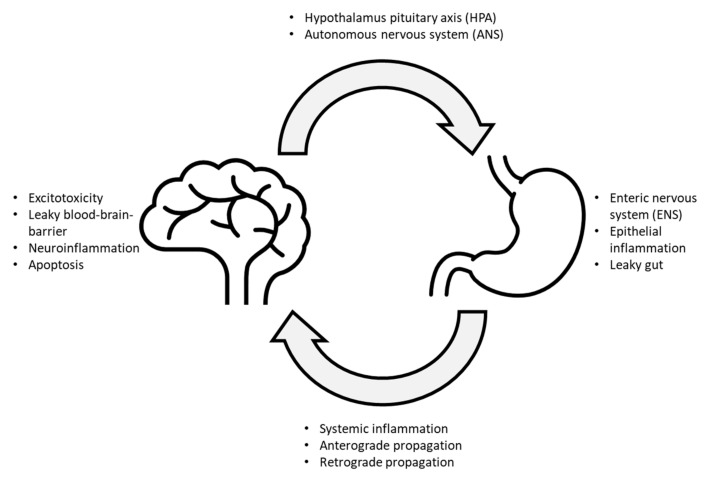
Summary of a potential gut–brain loop leading to a progressive development of neurodegenerative diseases.

Although the link between gut dysbiosis and neurological disorders remains speculative, anatomical evidence can now provide a clearer perspective of the bilateral communications between the gut and the brain. The microbiota–gut–brain axis is a recent description of a complex relationship between the gut epithelia, its associated microbes, and the brain. The central nervous system (CNS) and the enteric nervous system (ENS) are comprised of two terminal nodes, and the autonomic nervous system (ANS), sensory nerves, and the hypothalamic–pituitary–adrenal (HPA) axis serve as the edges that connect between the two nodes (Figure 1). In short, two major forms of communication routes are proposed to have facilitated microbial-associated pathogenesis of neurodegenerative disorders—the direct trans-neuronal route and blood transport through the systemic circulation. In the former, small infectious agents such as viruses can infect the cells and directly reach the brain through anterograde or retrograde transmissions. Other microbes can also induce or release prion-like proteins that can propagate along peripheral nerves to reach the brain. In the latter, microbial toxins and viruses can bypass intestinal epithelia, blood-brain barrier, and enters the brain through the systemic circulation. Viruses and toxins that successfully infiltrated into the systemic circulation can also cause inflammation at the local or systemic levels, leading to subsequent neuronal excitotoxicity, leaky blood-brain barrier, inflammation, and eventually cellular atrophy. In turn, the brain can also respond to these signals and influence the gut back through the ANS, ENS, or HPA pathways, either by direct neuronal stimulation or through the systemic release of hormones [5]. A comprehensive review of the relationship between the HPA, ANS, gut microbiome, and neurodegeneration was recently discussed by Peterson (2020) and Giordano (2020) [6,7].

It is currently recognized that alterations to the gut microbiome may precede many neurological and psychiatric symptoms and drive inflammatory diseases with unknown etiology. Gut-microbe alterations are associated with a wide range of neuropsychiatric and neurodegenerative conditions [6,8]. Furthermore, strict dieting conditions improve neurodegenerative phenotypes by modifying specific microbiome populations [9,10,11]. In the present review will focus on the potential impact of food on the microbiota–gut–brain axis. We will also examine how the resulting changes may be employed to treat neurodegenerative diseases. We will present early pathologies of AD and PD while highlighting evidence for microbe–gut–brain interactions, elaborate on relevant pathogenic microbe strains and review current and upcoming therapeutic strategies to manage gut dysbiosis in these diseases. Finally, we will discuss long-term diet plans and how dieting knowledge may manage microbial concentrations to prevent or treat neurodegenerative diseases.

## 2. Gut Microbiome Dysregulation in Neurodegenerative Conditions

### 2.1. Parkinson’s Disease (PD)

Idiopathic Parkinson’s disease (PD) is the second most common neurodegenerative disorder and characterized by four cardinal motor symptoms: resting tremor, bradykinesia, rigidity, and postural instability. The abnormal aggregation of the α-synuclein protein, typically involved in lipid-linked presynaptic vesicle trafficking and neurotransmitter release, results in the death of melanin-expressing dopaminergic neurons in the substantia nigra pars compacta of the midbrain [12]. Pathogenic α-synuclein may act in a prion-like fashion to form insoluble dysregulated proteasomal aggregates known as Lewy bodies that eventually results in neurotoxicity and midbrain dopaminergic neuron deaths [13,14]. Known genetic risks include autosomal dominant *SNCA* mutations and alterations to various mitochondrial genes, such as *LRRK2*, *DJ-1*, *Parkin*, and *PINK1* [15,16], all of which selectively impair metabolic pathways in dopaminergic neurons and induce neurotoxicity.

#### 2.1.1. PD Pathology

The early precedence of Lewy neurite formation in the midbrain and anterior olfactory nucleus led Braak to postulate that PD initiates from the olfactory epithelium and the GI epithelium [17]. Recent studies have also suggested that these early histological features are consistent with the preclinical non-motor symptoms, such as anosmia and constipation [18,19,20,21]. Constipation, in particular, was found to present in 61.4% of PD patients, of which about 24.5% had experienced discomfort in bowel movement before the onset of motor symptoms [22]. Moreover, early Lewy bodies were also present within enteric nerves and enteroendocrine cells (EECs) of the intestinal lumen [23,24,25], which further substantiates the claim that PD can originate from the gut. Together, these observations suggest that extrinsic factors from the gut could play a critical role in early sporadic PD pathogenesis.

Furthermore, PD is often purported to be a neuroinflammatory disease that arises from a highly oxidized (aerobic) gut environment. Fecal microbe transplant (FMT) of patient-derived PD microbiota showed shedding of bacterial endotoxins lipopolysaccharides (LPS) and short-chain fatty acids (SCFA) into the intestinal lumen [26]. Endotoxins such as LPS are detected by toll-like receptor 4 (TLR4) and nuclear factor kappa-light-chain-enhancer of activated B cell (NFκB) receptors on resident innate immune cells, such as the Paneth cells and macrophages of the mucosal epithelia [27,28]. In response to endotoxins, innate immune cells release pro-inflammatory cytokines, such as TNFα, IL1α, and IL6, leading to colitis and enhanced permeability of cytokines and endotoxins into the bloodstream [29]. Circulating endotoxins and cytokines can promote systemic inflammation and increase the permeability of the vasculatures within the liver, kidney, and brain, promoting α-synuclein deposition [30,31,32]. In the CNS, various cell types can also respond to the elevated inflammatory markers from systemic circulation; resident microglial cells become activated, astrocytes proliferate rapidly, and neurons become over-excitable [26,33]. Chronic exposure to inflammatory signals results in mitochondrial defects, formation of oxidative radicals, activation of apoptotic or necrotic pathways, and thereby leads to excitotoxicity and cell death. Clinical studies also support the inflammatory-based hypothesis, with immunosuppressants ameliorating PD pathogenesis [34,35], and with deficiency of CD4+ T-cells correlated to markedly decreased dopaminergic neuronal loss in MPTP mouse models [36]. In the gut, abnormal folding of α-synuclein and α-synuclein aggregates can also result in the release of pro-inflammatory cytokines [37,38]. Gut injected α-synuclein fibrils were shown to propagate to the midbrain in mouse models, while vagotomy prevented parkinsonism symptoms [39].

#### 2.1.2. Microbes Associated with PD

In general, there is a collective shift in the distribution of the gut microbiome to favor a pro-inflammatory environment that encourages the development of protein aggregation and neurodegeneration. Recent studies have also begun to reveal specific bacterial-induced pathways that may play a more direct role in PD pathogenesis. Here, we classify candidate bacterial microbes into two broad categories: those that increased with PD and those that decreased with PD. Bacteria found with higher prevalence in a PD gut are more likely to secrete endotoxins that can induce inflammation and facilitate methanogenesis. The heightened expression level of the LPS gene was reported in the PD fecal microbiome [40]. Examples of bacteria species that are increased in abundance in PD patients include Enterobacteriaceae, Akkermansia, Catabacter, Oscillospira, Lactobacillus, and Bifidobacterium species [41]. In particular, certain species from the Gram-negative Enterobacteriaceae that comprises common strains such as Escherichia coli, Klebsiella, Salmonella, Shigella, and Yersinia pestis were shown to secrete pro-inflammatory LPS from their cell walls and were positively correlated with the severity of motor symptoms such as postural instability and gait difficulty [42,43]. The monocolonization of curli-producing Escherichia coli enhances α-synuclein pathology through the production and association of bacterial amyloid protein [44].

Intriguingly, a recent meta-analysis also highlighted an increase in methane-producing bacteria such as the *Christensenella* spp. and *Methanobrevibacter* in PD patients [45]. Methane is produced either through the metabolic pathway of acetate degradation or from methanogenesis from H_2_ and CO_2_ with the aid of the cofactors coenzyme B and M [46]. An increase in methane production increases intraluminal pressure and reduces peristaltic movements, resulting in constipation [46,47]. Consequently, bacterial populations decreased in PD patients are more likely to be anaerobic, partaking in anti-inflammatory and antioxidative pathways. The Prevotellaceae family is one of the mucin-producing commensals that secrete short-chain fatty acids (SCFAs) from the fermentation of dietary fibers such as butyrate [48]. To further prove the point, the transplantation of fecal matter from PD patients into the guts of germ-free mice has been linked to SCFA alteration, α-synuclein aggregation, and movement dysfunction [26]. Reduction of SCFAs leads to increased gut permeability to bacterial or microbial toxins. Studies on patient stools also observed a decrease in specific populations of anaerobic microbes involved in butyrate production. These include *Roseburia*, *Blautia*, *Faecalibacterium*, and *Dorea* [49]. Butyrate is an anti-inflammatory and antioxidant component of dietary fibers, which elicits its effect by inhibiting the NFκB pathway [50], increasing both colonic glucagon-like peptide-1 (GLP-1) and GLP-1 receptor levels [51], acting through the enteroendocrine pathways, and initiating epigenetic mechanisms to elevate anaerobic bacterial levels. Decreased butyrate concentration is associated with increased oxygen levels in the gut mucosa, may enhance oxidative bacterial species, and heightens the risk of oxidative damage to the gut. However, these findings are in stark contrast to an opposing study which suggests that butyrate may exacerbate MPTP-induced PD through NFκB-independent pathway(s) [52]; suggesting that complex microbe–microbe and host–microbe interactions may also play an important role in regulating inflammatory pathways at the gut.

Careful consideration must still be applied to differentiate the effects of microbial changes due to disease progression or from preexisting pharmacological treatment. *Helicobacter pylori* is an infamous inflammation-inducing microbe known to be involved in peptic ulcers and gastric adenocarcinoma development [53,54]. Gut levels of *H. pylori* are enhanced in both acidic gastric mucosa and the basic intestinal environment of PD patients. However, the link between *H. pylori* and PD may involve its role in hindering the absorption of L-Dopa into the systemic circulation [55]. In turn, this may indicate that the fitness and prevalence of this bacterial species could be associated with long-term L-Dopa intervention.

The role of other microbes, such as archaea, fungi, and viruses, in the pathogenesis of PD, has also been left largely unexplored. Viruses are likely pathological agents that could induce PD through direct infection of CNS neurons or enter from the systemic circulation through interactions with the PNS neurons or the BBB [56]. Viruses of different genetic origins were associated with parkinsonism-like symptoms, including DNA virus families such as *Herpesviridae* and RNA virus families such as *Flaviviridae*, *Picornaviridae*, *Paramyxoviridae*, *Orthomyxoviridae*, *Retroviridae*, and *Rhabdoviridae* [57]. The type A influenza virus from the *Orthomyxoviridae* family is one prominent example. In the 1918 influenza pandemic, parkinsonism symptoms emerged after the widespread viral encephalopathy [58,59]. However, it has now been shown that systemic infection of Influenza A viruses such as H1N1 promotes CD8⁺ T cell death and reduces cytokine production through the programmed death-ligand 1 (PD-L1) pathway [60], which suggests that reduced gut viral load may enhance inflammation and therefore parkinsonism. From the *Retroviridae* family, the human immunodeficiency virus (HIV) primarily infects CD4+ cells, escalating to acquired immunodeficiency syndrome (AIDS), and can bypass the BBB from systemic circulation [61]. Alternatively, neuron-infecting viruses, such as rabies viruses from the *Rhabdoviridae* family, polio [62], measles [63], herpes, and the recent SARS-Cov-2 [64], are purported to avoid the BBB completely, either anterogradely or retrogradely through trans-neuronal routes [65]. Additionally, these viruses require specific adsorption factors on both the viral capsid/membrane and the neuronal surfaces and specific infections of aminergic neuronal populations. However, it is noteworthy that increased viral loads are also associated with partial or severe immunodeficiency, which can reduce the occurrence of inflammatory PD. An overall decrease in virus count in PD patient stools suggests opposing and protective effects against other PD-causing microbes in the gut [66]. Nevertheless, more research must be undertaken to shed light on the viral induction mechanism in the development of parkinsonism and its potential roles in causing synucleinopathy.

#### 2.1.3. Current Evidence for Microbe-Related Treatment for PD Patients

Knowledge of the healthy composition of gut microbes sets the pace for the pre-emptive prevention or adjuvant treatment of PD. However, current clinical studies that involve fecal transplantation are limited. Exposure to certain types of broad-spectrum antibiotics, such as tetracyclines, and antifungal medications have led to an elevated risk of PD [67]. One case study of FMT for profound dysbiosis in PD patients resulted in defecation and recovery from tremors in the legs, but the beneficial impacts only persisted for a week [20]. Oral butyrate consumption has also been shown to be beneficial for reducing inflammatory activity and cytokine levels of circulating monocytes in obese patients [68]. Another explored strategy is to target specific microbial genes for adjuvant treatment. For example, a prokaryotic homolog of the tyrosine decarboxylase enzyme present in the common bacterium *Enterococcus faecalis* of certain patients prematurely converts L-dopa into dopamine in the gut, thereby reducing the dopamine available to the brain. Co-treatment of L-dopa with aromatic l-amino acid decarboxylase inhibitor carbidopa reduces gut metabolism by tyrosine decarboxylase and improves treatment outcomes [69]. Another potential clinical usage includes using gut microbes as potential early biomarkers.

### 2.2. Alzheimer’s Disease (AD)

AD is currently the world’s most common neurodegenerative dementia and is characterized by progressive loss of memory, confusion, and decline in other cognitive functions. The disease is characterized by progressive shrinkage of the medial temporal lobe in the earlier stages, resulting in characteristic retrospective short-term memory loss. In the late stages, atrophy of the neocortex results in cognitive decline and permanent personality changes [70]. Like PD, AD is usually characterized into early or late-onset differentials, with early-onset forms associated with family history and late-onset forms considered idiopathic and sporadic. Genetic risk factors for familial AD include mutations to amyloid precursor protein (*APP*), *PSEN-1/2*, tau proteins, and tau kinase genes, while sporadic AD is highly correlated with elevated expression profiles of certain mutant apolipoprotein E (APOE) isoforms, more specifically the APOE4 mutations [71]. However, sporadic AD can also be associated with vascular dementia, likely due to aggregations of damaged proteins occurring in microinfarcts arising from vascular thrombosis [72,73]. As such, unhealthy behaviors such as sedentary lifestyles and high-fat Western diets predispose patients to a higher risk of acquiring AD through chronic conditions such as hypertension and hypercholesterolemia.

#### 2.2.1. AD Pathology

AD is regarded as a form of proteinopathy driven by the abnormal accumulation of extracellular amyloid-β (Aβ) plaques and intraneuronal neurofibrillary tangles involving tau protein [74,75,76,77,78,79]. However, there have been failures in numerous high-profile clinical trials to reduce Aβ plaque formation and tau accumulation, with read-outs measured by behavioral improvements, PET scans, and peripheral ELISA tests. The failures question the accuracy of the amyloid and tau theory of AD development.

Although aducanumab has been recently approved by the FDA for AD treatment, there has been much scrutiny of the validity of the evidence presented and its potential clinical effect [80]. Furthermore, the most promising Aβ-targeting monoclonal antibodies, with a similar mechanism of action and aims to aducanumab, such as solanezumab, bapineuzumab, crenezumab, and gantenerumab, have failed their respective Phase III trials [81,82]. The role of normal physiological levels of soluble and insoluble Aβ has also been, at best, contradictory. Large doses of Aβ aggregates cause significant presynaptic and postsynaptic defects, but a low dosage of Aβ to hippocampal neurons promotes neuroprotective BDNF release. On the other hand, tau therapies in ongoing clinical trials involving the use of small-molecule drugs and small interfering RNAs (siRNAs), that can modify phosphatase and kinase activity (e.g., memantine), inhibit tau modifications, and aggregation [83], have had limited success. As such, research into other pathological mechanisms is also in progress, with an increasing focus on metabolic and physiological processes such as mitochondrial dysfunction, insulin resistance, neuroimmunomodulation, and cerebral hypoperfusion. Nonetheless, there are no satisfactory explanations that can fully describe the initiation and pathophysiology of AD, suggesting that external influences such as pathogens, iron, manganese, other heavy metals, and oxidizing aerosols could also play vital roles in AD pathogenesis [84].

The microbiota–gut–brain axis could be a vital link in describing the early development of Aβ aggregation in early sporadic AD. In particular, the inflammation-based hypothesis suggests that monomeric Aβ is first released from the prevailing inflammation in the gut and therefore enters systemic circulation, bypassing the leaky gut endothelium. The accumulation of Aβ peptides in the brain propagates in a prion-like fashion to form amyloid fibrils. Therefore, the formation of amyloid plaques triggers neuroinflammation, influencing neurofibrillary tangle formation, activating CD33 and TREM2 pathways in resident microglia [85], and therefore leading to subsequent excitotoxicity and neurodegeneration.

#### 2.2.2. Microbes Associated with AD

Much like in PD, similar microbes responsible for gut inflammation, mucin-degradation, and oxidative damage are elevated in AD patients. These include the *A. muciniphila*, *Bacteroides*, and certain species from the Enterobacteriaceae family which are known to release LPS [86,87]. However, unlike in PD, studies have further identified certain bacterial species that might even participate directly in amyloid genesis. The common coliform bacteria *E. coli* from the Enterobacteriaceae family may secrete a bacterial homolog of Aβ to form biofilm for bacterial communications and protection from antibiotics and host defenses [88,89,90]. Although the evolutionary history and primary structure of mammal and bacterial amyloids are different, bacterial amyloids might still cause a cross-seeding effect in the host, in which one amyloidogenic protein (for example the curli of *E. coli*) or sub-domain of the protein (beta-pleated structure of curli) serves as a scaffold for aggregation of other amyloidogenic proteins such as tau, α-syn, prion, or amyloid Aβ [91].

Besides directly triggering aggregation, exposure of bacterial amyloid proteins to the gut may cause priming of the immune system, enhancing immune response to endogenous production of CNS amyloidosis via TLR 2/1, CD14, NFκB, and iNOS [92,93,94]. Studies involving germ-free mouse models of AD further support the hypothesis that the neuroimmunology-dependent removal of Aβ is modulated by the gut microbes since the depletion of gut bacteria results in an increased microglial uptake of Aβ [95] and the FMT of human AD patient fecal samples into germ-free C57BL/6N mice recapitulated memory-related behavioral deficits [96]. Similar to PD, downregulated bacterial colonies include the mucin-producing Prevotellacae, and anti-inflammatory butyrate-producing bacteria such as *Blautia*, *Coprococcus*, *Roseburia*, *Faecalibacterium*, and *Dorea* [87,97]. As previously mentioned, Prevotellacae secretes SCFA, which reduces inflammation and preserves the integrity of gut intestinal barriers [48], while butyrate-producing bacteria play a vital role in preventing and reducing NFκB pathways and may help to reduce localized inflammation and oxidative species in the gut. Interestingly, the removal of the *Lactobacillus fermentus* bacterial strain is associated with memory deficits observed with the consumption of ampicillin [98].

#### 2.2.3. Current Evidence for Microbe-Related Treatment of AD

Early microbe-based probiotics treatments for AD have led to contradictory outcomes. Supplementation of probiotics from the *Lactobacillus* and *Bifidobacterium* genera was ineffective in alleviating cognitive decline and serum biomarker levels in AD patients (*n* = 23) [99]. However, another similar small-scale clinical trial (*n* = 20) using a *Lactococcus lactis* to *Lactobacillus* and *Bifidobacterium* mixture was reportedly able to alter gut microbiome composition and alleviate AD symptoms by affecting the tryptophan metabolic pathway [100]. The same study also suggested that probiotic concoction enhanced levels of *Faecalibacterium prausnitzii*, a butyrate-producing strain associated with anti-inflammation in Crohn’s disease [101], while inflammation-associated markers such as zonulin were decreased [100].

Perhaps the occurrence of AD is indeed triggered by an increasing prevalence of pathological microbes in the gut. In germ-free APP transgenic mice, the absence of gut microbiota reduced cerebral Aβ amyloid pathology compared to control mice with an unaltered intestinal microbiota [102]. Caloric restriction reduces the level of the *Bacteroides* colonies that exacerbate Aβ deposition [103]. Similarly, an antibiotic cocktail induced alteration to the gut microbiome of APPSWE/PS1ΔE9 and APPPS1-21 mouse models, which led to a reduction in Aβ deposition in the brain. However, this effect was absent when individual antibiotics were administered [104], suggesting that widespread gut dysbiosis in AD patients is more likely to perturb specific metabolic pathways and disturb normal interspecies mutualism. Alterations to gut microflora might have caused the increase in pro-inflammatory bacterial species and resulted in localized inflammation. Hence, conducting FMT with a fecal sample containing more than one probiotic strain specific to AD may increase the chances of success of the FMT. In all, a summary of the points discussed in AD (and PD) can be found in Table 1.

## 3. Impact of Diet on Gut Microbiome Composition

Extrinsic influences from our daily lifestyle are likely to be major factors in the pathogenesis of AD and PD. Although the underlying etiologies are still under scrutiny, special attention can be given to lifestyle factors, such as exercising, dieting, smoking, and drinking habits, which may influence gut microbiota and worsen or ameliorate the symptoms and neurodegenerative diseases progression. Our diet is an important aspect of our daily lifestyle and can be controlled; the proportion of ingested dietary nutrients such as proteins, carbohydrates, fats, and dietary fibers can directly or indirectly alter metabolic and transcriptional profiles in both the host and their microbes, affecting downstream metabolic activities and playing a significant role in the regulation of host physiology [105]. For example, a low-fiber diet may trigger the expansion of mucus-degrading bacteria such as *A. muciniphila* and *Bacteroides caccae,* which can lead to leaky gut and colitis [106]. A potential method of mitigating dysbiosis is to adopt a more well-rounded diet with sufficient nutrients to promote a healthy and diverse gut microflora.

### 3.1. Diet(s) That Are Positively Associated with Neurodegenerative Diseases—Current Evidence for Microbe-Related Treatment of AD

The Western-style diet (WD) is a lifestyle associated with a disproportionately high amount of processed food and loaded with excessive caloric content, fats, proteins, and carbohydrates, but with an insignificant number of vitamins and dietary fibers. Examples of WD include high sugar food and beverages, fast food, and deep-fried and processed meat. High sugar content and processed food are implicated in gut inflammation and increased incidence of bowel-associated disorders, such as inflammatory bowel syndrome (IBS) and colorectal cancer. WD can interfere with the generation of short-chain fatty acids (SCFA) [107] through dietary fiber deficiency. The low fiber consumption in WD drives microbes to use alternative sources of nutrients, such as amino acids or dietary fats for energy. This, in turn, reduces the fermentative ability of the microbes and SCFA as they are minor end products [108]. In addition, a high-fat diet also spurs an increased production of bile acids (deoxycholic and lithocholic acid), which generally reduces the survival of bacteria that prefer plant-based diets (*Firmicutes* and *Proteobacteria* phyla dominate, *Prevotella* enterotype) [109], while promoting the growth of bile-resistant bacteria which are more pro-inflammatory, such as the *Bilophila wadsworthia*, *Alistipes putredinis*, and *Bacteroides* species [110]. Refined sugar consumption, such as in the form of sugar-sweetened beverages, was also linked to a reduction in microbial diversity [111]. Recent preliminary evidence has also shown the appearance of neurological syndromes with changes in diet, which are associated with gut dysbiosis and an overall reduction in gut microbiome diversity [112].

### 3.2. Diets That Are Negatively Associated with Neurodegenerative Diseases

#### 3.2.1. Ketogenic Diet (KD)

The ketogenic diet (KD) comprises a low carbohydrate, moderate portion of protein, but high-fat diet. Ketosis occurs due to β-oxidation of SCFA, monounsaturated fatty acids (MUFAs), and PUFAs within the liver, in the absence of carbohydrates and glycogen. The major difference between KD and a high carbohydrates diet is that the former favors the production of ketone bodies such as acetoacetate and β-hydroxybutyrate (BHB) that can act as an alternative energy source. As an energy source, ketone bodies can be directly metabolized (ketosis) into acetyl-CoA and fed into the TCA cycle to drive energy production or act in other measures, such as regulating protein activity and influencing gene transcription through β-hydroxybutyration to reduce systemic inflammation [113]. KD was first adopted clinically to treat refractory epilepsy in children and adults and was hypothesized to play a neuroprotective role in the brain [114,115]. Current evidence, therefore, suggests KD as an appropriate lifestyle for weight loss and diabetic management against hyperinsulinemia [115] and with potential usage in PD.

However, little is known about if ketosis affects gut dysbiosis and if gut microbe alterations by long-term KD can ameliorate the symptoms of early AD and PD. Acute short-term changes to the gut microbiome are likely to be fueled by a shift from carbohydrate-rich meals to high triglycerides content within the intestinal lumen, while long-term production of the ketone bodies drives gut microbial shifts distinct to high-fat diets. Examples of bacteria species elevated through ketogenic diet include the mucin-degrading *A. muciniphilia* and the probiotic *Lactobacillus*, while *Bifidobacterium*, sulfate producing *Desulfovibrio*, and *Turicibacter* are reduced [116]. The high levels of BHB released by epithelial cells into the intestinal lumen lower *Bifidobacterium* count, which inhibits Th17 helper cells and reduces the possibility of local epithelial inflammation and leaky gut syndrome [117].

#### 3.2.2. Mediterranean Diet

Mediterranean diet (MD), which is considered a healthy balanced diet, involves not only plant-based foods, but also includes greater consumption of unsaturated fats primarily from olive oil, low to moderate consumption of dairy products, and a moderate to high consumption of fish [118]. While WD increases the occurrence of gut dysbiosis, healthy dietary patterns favorably modulate the gut microbiome [105]. MD has been associated with a greater abundance of *Faecalibacterium prausnitzii*, *Eubacterium eligens*, and *Bacteroides cellulosilyticus*, all of which are major SCFA producers [119]. Specifically, selected nutrients within MD are known for these roles. The high fiber content provides more fermentable fibers in the gut favoring SCFA-producing bacteria such as *Prevotella* and *Lactobacillus*. In addition, the presence of these bacteria has also been correlated with reduced *Escherichia*, *Turicibacter*, and *Akkermansia*. *Escherichia* and *Turicibacter* are associated with increased gut inflammation, and *Akkermansia* reduction aids the degradation of the mucosal barrier within the gut, hence reducing the risk of pathogenic infections [120]. The type of dietary fat consumed also modulates the gut microbiome. Olive oil consumption, which is rich in omega-6 polyunsaturated fatty acids (n6-PUFA), has been linked to increased SCFA production [121], while a greater intake of n6-PUFA is inversely associated with Alzheimer’s disease onset [122]. Omega-3 polyunsaturated fatty acids (n3-PUFA) obtained from fish consumption have also gained significant attention for their ability to moderate gut communities. For example, n3-PUFA is mainly metabolized by *Bifidobacterium* and *Lactobacillus*, both of which are known to contribute beneficial effects [123] and improve the gut environment, while minimizing the growth of *Enterobacteria*. They improve the intestinal mucus barrier and reduce the occurrence of low-grade inflammatory responses within the intestine.

#### 3.2.3. Plant-Based Diet

Plant-based diets comprise mainly fibers and complex carbohydrates, with a particularly high count of antioxidants, vitamins A, C, E, and phytochemicals, while being generally low in fat and simple sugars. Plant-based diets can vary from a full vegan diet consisting of non-animal products, such as vegetables, grains, nuts, and fruits, to more lenient variants that selectively exclude meat, poultry, and seafood but may include dairy products and eggs. Although the exact health benefits of plant-based diets remain unclear, various ethnic groups eating variations of plant-based diets were found to have substantially lower PD prevalence [124].

Out of the multitude of nutrients in plant-based products, soluble and insoluble dietary fiber remain as one of the most significant. Dietary fiber cannot be digested by humans, but certain gut-associated bacteria populations might be able to utilize fiber as a food source. For general human health, an increase in dietary fiber content poses many metabolic and health benefits, including feelings of satiety and aiding in preventing the overconsumption of food. For the gut physiology, dietary fibers play an important role in modulating the pH of the gut and elevating levels of the probiotics of *Bifidobacterium* and *Lactobacillus* in the intestines [125]. Moreover, dietary fibers are also associated with an increase in levels of SCFAs such as butyrate and its isoforms. As explained in previous sections, this, in turn, is associated with an increase in butyrate-producing bacteria, which help to alleviate inflammation and prevent the growth of pathogenic, pro-inflammatory strains.

Antioxidants can also be found in high amounts in fruits and vegetables, namely citrus, berries, and dried fruits. Higher endogenous levels of antioxidants, such as glutathione and ascorbic acid (Vitamin C), led to better prognostic outcomes in children with severe malnutrition [126], probably by allowing anaerobes to thrive in a more oxidizing and aerobic environment. Antioxidants may also directly aid in reducing the impact of oxidative stress, neutralizing reactive oxygen species (ROS), and may help ameliorate local and systemic inflammation in PD and AD. Supplementing uric acid, ascorbate, and glutathione into bacterial culture media enhanced the butyrate production associated with reduced inflammation in the gut [127].

Phytochemicals, commonly found in fruits, vegetables, grains, coffee beans, and tea leaves, provide beneficial effects for reducing gut dysbiosis. A recent randomized controlled trial with phytochemicals showed improvements in the gut microbiome from an increased abundance of the probiotic Ruminococcaceae, associated with hepatic metabolism and immune function, following a selective vegetarian diet in PD patients [128,129]. Polyphenols found in berries and tea are examples of phytochemicals shown to increase gut microbiome diversity. Gut concentrations of *Faecalibacterium prausnitzii*, a known anti-inflammatory bacterium, were observed to be positively correlated with increased polyphenol intake [111]. Other polyphenols such as flavan-3-ols, anthocyanins, tea flavonoids, and isoflavones have also been associated with a significant increase in the *Clostridium coccoides-Eubacterium rectale*, *Lactobacillus*, and *Bifidobacterium* clusters [130,131,132]. A meta-analysis by Ma and Chen (2020) asserted the benefits of polyphenol consumption, citing a significant increment of *Lactobacillus*, *Bifidobacterium,* and a reduction in pathogenic *Clostridium* species [133]. In turn, these bacteria facilitate the metabolism of polyphenols, which are converted to phenolic acids, counteracting oxidative stress, while aiding the regulation of tight junction proteins for improved intestinal permeability [134]. However, it is unclear if a plant-based diet is also beneficial due to the removal of neurotoxic components from a regular urban diet. For example, diets high in animal fat or cholesterol are associated with a substantially increased in risk of PD, but not food prepared with plant-derived fat [124].

#### 3.2.4. Caloric Restriction Diet (CR)

Caloric restriction (CR) is a group of dietary interventions in which an individual reduces the uptake of caloric content without malnutrition or deprivation of essential nutrients. The caloric content for a CR meal can be reduced from a recommended daily amount of 1600 calories to 800 calories for females, and from 2500 calories to 1500 calories for males [135]. The implementation of CR may vary from continuous restriction by actively reducing the amount of food intake per meal, to the highly popularized time restriction, where consumption is only allowed within a daily eating window of 6–10 h. Interestingly, an intermittent diet of three to twelve months in animal models helped weight loss, regardless of fasting duration and frequency [136].

Increasing evidence from association and laboratory studies has shown that CR can provide tangible benefits for patients with neurodegenerative diseases. First, a study on a female Tg2576 familial AD mouse model showed reduced *Bacteroides* colonization, which downplays the effect of Aβ deposition [103]. In contrast, CR increases the prevalence of probiotics, such as the anti-inflammatory *Lactobacillus* strains [11,137]. Moreover, microbiome modulation via CR is related to reduced circulating LPS and low-grade inflammation in the gut [11]. CR has also been shown to protect the dopaminergic neurons of the midbrain from neurotoxins in mice. In the stomach and duodenum, heightened transcriptomic expressions of hsp70 were assessed to play a partial role, especially in protecting rat pheochromocytoma PC12 cells from Aβ peptide toxicity [138,139]. CR is reportedly also able to increase Sirtuin1 expression, to regulate the gut microflora and metabolites [140]. Most importantly, CR limits food intake and improves BMI, cholesterol levels, and blood pressure in both obese and non-obese adults, and significantly lowers the risk of AD and vascular dementia through altering the gut compositions of microbial species from the *Clostridium*, *Dorea*, *Coprococcus, Clostridiales*, *Streptococcus,* and *Mobiluncus* genera [141].

**Figure 2:**
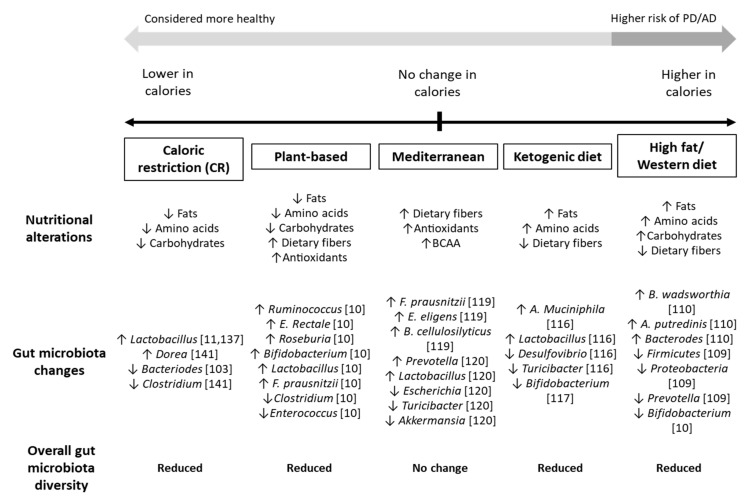
Influence of different restrictive diets on the gut microbiota. High fat and high sugar diets, such as the Western diet (WD), are likely to lead to increases in inflammatory-associated microbes within the gut. Diets rich in dietary fibers, branched chain amino acids (BCAA), and antioxidants are associated with an increase in probiotic strains, which may reduce the risk of and ameliorate neurodegenerative conditions. Strict diets, such as caloric restriction, plant-based, and ketogenic diets typically resulted in a loss of gut microbiota flora, which may lead to dysbiosis in the long run. Overall, a well-balanced diet with sufficient, rounded nutrition is considered the most suitable for managing AD and PD.

However, some evidence does argue against CR as a plausible dietary practice for managing AD and PD. A recent study on grey mouse lemurs showed that CR accelerated loss of grey matter in the cerebrum, although the animals’ life spans were extended by 50% without cognitive benefits [142]. Prolonged CR is also associated with decreased gut microbiota diversity, reduced core temperature and basal metabolic activities, and fatigue [143]. In summary, the benefits of CR are likely to vary depending on an individuals’ disease pathology and status, and likely to be more beneficial for patients that made the switch from unhealthy WD-like diets and sedentary lifestyles (Figure 2).

## 4. Discussion

The precise etiology of many neurodegenerative diseases remains unclear. In the past decades, Braak and colleagues’ anatomical works have caused a rising awareness of the role of microbiota in influencing the gut–brain axis and that they may be causative agents of neurodegenerative diseases. In this review, we covered numerous studies spanning a diversity of microbial strains affected in AD and PD. However, the exact causative links between various pathological strains and neurodegenerative diseases remain a mystery; with no single archaea, bacterium, virus, or fungus species solely determined as a causative agent(s). As clearly seen from animal experiments, adding or removing a single microbe species is insufficient for seeding the diseases, and neither did the addition nor removal of a single species from AD/PD guts help in ameliorating the diseases. Most FMT clinical trials, therefore, focused on an established probiotic mix, typically containing culturable probiotics strains such as *Lactobacillus* and *Bifidobacterium* [144,145,146,147]. However, their findings have yet to reveal any desirable outcomes significant enough to be translated into clinical practice.

Perhaps in many of these studies we still have an imperfect knowledge of the functions of individual microbes in the gut. Of the microbes highlighted, the probiotic *Lactobacillus* family stands out as one of the most controversial, having its proportions upregulated in both AD and PD guts, but also elevated through CR and WD diets. Common *Enterococcus* strains such as *E. coli* facilitate digestion and are present within healthy individuals, but are also reported to release LPS and potentially secrete bacterial forms of amyloid, aiding in pathogenesis [148]. This evidence suggests that other factors such as genetic and epigenetic regulations, rather than solely species identification, may play a more prominent role in the pathogenesis of AD and PD.

Several possible reasons may account for these heterogeneous findings. Horizontal gene transfer, or the transfer of genetic material between microbes, is a plausible mechanism that can explain the complicated pathologies. *E. coli* strains can acquire virulence factors through transposons, conjugation, enterotoxins, and bacteriophages [149]. For the majority of the AD and PD patients enrolled in clinical trials, concurrent therapies also pose an additional confounding issue in determining precise microbiota diversity and the efficacy of FMT. As previously mentioned, prolonged high-dose L-Dopa administration may create a strong selection pressure and therefore promote the survival of L-Dopa metabolizing species [150]. Together, these findings suggest that the interplay of many factors, possibly involving genetics, epigenetics, metabolic, immune regulations, and drug interactions, could have influenced the outcome of the studies. Future research in the field should take these factors into special consideration and create mixed host–microbe and microbe–microbe co-cultures to segregate pathogenic factors from other confounding factors.

With such complex microbe–microbe, host–microbe, drug–microbe, and drug–host interactions, it is difficult to pinpoint specific dietary elements or strict dieting regimens that may help prevent or ameliorate AD and PD progression. Nevertheless, a few dietary risk factors are prominent. High salt, high fat, and high sugar diets, such as the WD, subjects the patient to inflammation at the gut level and are positively associated with hypercholesterolemia and hypertension. On the other hand, restrictive diets such as CR, plant-based diets, and KD may contain higher amounts of dietary fibers, antioxidants, polyphenols, and ketone bodies, and have been reported to have anti-inflammatory properties, help to reduce oxidative stress, and facilitate smoother bowel motion. Interestingly, AD patients show better outcomes with treatments that decrease gut microbe diversity. Therefore, long-term CR and a plant-based diets that reduce gut biodiversity would likely be beneficial for AD patients. In contrast, a treatment that increases gut biodiversity in PD patients often appears to ameliorate gut dysbiosis and promote better peristaltic bowel movements. FMT treatment with a well-balanced diet and a high dietary content, therefore, seems more promising for PD patients.

Notably, special attention must also be placed on the prevailing lifestyles of chronic neurodegenerative patients on top of their diet. Especially in AD involving vascular dementia, other health indicators such as BMI, cholesterol, and blood pressure should be finely regulated. A restrictive diet may play a vital role for these patients in alleviating health risks and CVD factors. However, these highly restrictive diets also predispose patients to a higher risk of malnutrition through decreased gut microbiota diversity. As such, a well-balanced diet for AD and PD should ideally comprise of recommended daily caloric intake, but also with an increase in anti-inflammatory nutrients such as dietary fibers, lean proteins, low unsaturated fats, a higher concentration of complex sugars, antioxidants, vitamins, and water content. Such a diet, for example the MD, is more likely to be associated with increased gut microbiota flora and a healthy gut, without compromising on the calories required for sustaining regular metabolic activities. Recently, media platforms have also begun to popularize complex diet schedules, such as adopting intermittent fasting in the short run and converting to MD in the long run. However, there is limited evidence of the efficacy of such treatments.

Finally, pairing healthy dietary remedies with other forms of intervention (e.g., fecal microbiota transplant, enema, exercise, and probiotics) may influence the gut microbiome and attenuate the development of neurodegenerative diseases [129,151,152]. Exercises was also shown to enhance neuroprotective brain-derived neurotrophic factor (BDNF) levels in the brain [153,154]. Perhaps psychological interventions such as cognitive-behavioral therapy and mindfulness may also induce neuronal stimulation or alter the hormonal levels that influence the gut microbiota [155,156,157,158]. Other psychological and social approaches remain prospective in regulating gut microbial concentrations and ameliorating neurodegenerative diseases. In general, a healthy mind with balanced diet is likely a better prevention or reduction of symptoms in AD and PD patients.

## Figures and Tables

**Table 1:** Bacterial microbial species that were either upregulated or downregulated, as well as their associated pathways in the pathogenesis of AD and PD.

Disease	Upregulated/Downregulated	Mechanistic Pathway	Microbe Species
PD	Upregulated	Pro-inflammatory (Secrete LPS)	*Lactobacillus* [41] *Akkermansia* [41] *Bifidobacterium* [41] Enterobacteriaceae [42,43]
		Methane production	*Christensenella* spp. [45] *Methanobrevibacter* [45]
		Bacterial amyloid production	*Escherichia coli* [44]
	Downregulated	Anti-inflammatory (Secrete SCFA)	Prevotellaceae [48]
		Butyrate production	*Roseburia* [49] *Faecalibacterium* [49] *Blautia* [49]
		Mucin-degrading	*Dorea* [49]
AD	Upregulated	Pro-inflammatory (Secrete LPS)	*H. pylori* [86] *A. muciniphila* [86] Enterobacteriaceae [86,87] *Lactobacillus* [86] *O. splanchnicus* [87] *B. fragilis* [87]
		Bacterial Aβ crosslink	*Klebsiella* spp. [87] Enterobacteriaceae, *E. coli* [88,89,90]
		Mucin-degrading	*Dorea* [97]
	Downregulated	Anti-inflammatory (Secrete SCFA)	Prevotellaceae [87]
		Butyrate production	*Clostridium* [87] *Coprococcus* [87] *Roseburia* [87] *Faecalibacterium* [87]
		Modulation of gut microbiome profile	*Lactobacillus fermentus* [98]

## Data Availability

Not applicable.

## References

[1] Jandhyala S.M., Talukdar R., Subramanyam C., Vuyyuru H., Sasikala M., Nageshwar-Reddy D. (2015). Role of the Normal Gut Microbiota. World J. Gastroenterol..

[2] Nagpal R., Mainali R., Ahmadi S., Wang S., Singh R., Kavanagh K., Kitzman D.W., Kushugulova A., Marotta F., Yadav H. (2018). Gut Microbiome and Aging: Physiological and Mechanistic Insights. Nutr. Healthy Aging.

[3] Xu L., Surathu A., Raplee I., Chockalingam A., Stewart S., Walker L., Sacks L., Patel V., Li Z., Rouse R. (2020). The Effect of Antibiotics on the Gut Microbiome: A Metagenomics Analysis of Microbial Shift and Gut Antibiotic Resistance in Antibiotic Treated Mice. BMC Genom..

[4] Budi N., Safdar N., Rose W.E. (2020). Treatment Issues in Recurrent Clostridioides Difficile Infections and the Possible Role of Germinants. FEMS Microbes.

[5] Duran-Pinedo A.E., Solbiati J., Frias-Lopez J. (2018). The Effect of the Stress Hormone Cortisol on the Metatranscriptome of the Oral Microbiome. NPJ Biofilms Microbiomes.

[6] Ceppa F.A., Izzo L., Sardelli L., Raimondi I., Tunesi M., Albani D., Giordano C. (2020). Human Gut-Microbiota Interaction in Neurodegenerative Disorders and Current Engineered Tools for Its Modeling. Front. Cell. Infect. Microbiol..

[7] Peterson C.T. (2020). Dysfunction of the Microbiota-Gut-Brain Axis in Neurodegenerative Disease: The Promise of Therapeutic Modulation with Prebiotics, Medicinal Herbs, Probiotics, and Synbiotics. J. Evid. Based Integr. Med..

[8] Zhu S., Jiang Y., Xu K., Cui M., Ye W., Zhao G., Jin L., Chen X. (2020). The Progress of Gut Microbiome Research Related to Brain Disorders. J. Neuroinflamm..

[9] Włodarek D. (2019). Role of Ketogenic Diets in Neurodegenerative Diseases (Alzheimer’s Disease and Parkinson’s Disease). Nutrients.

[10] Tomova A., Bukovsky I., Rembert E., Yonas W., Alwarith J., Barnard N.D., Kahleova H. (2019). The Effects of Vegetarian and Vegan Diets on Gut Microbiota. Front. Nutr..

[11] Zheng X., Wang S., Jia W. (2018). Calorie Restriction and Its Impact on Gut Microbial Composition and Global Metabolism. Front. Med..

[12] Man W.K., Tahirbegi B., Vrettas M.D., Preet S., Ying L., Vendruscolo M., De Simone A., Fusco G. (2021). The Docking of Synaptic Vesicles on the Presynaptic Membrane Induced by α-Synuclein Is Modulated by Lipid Composition. Nat. Commun..

[13] Ma J., Gao J., Wang J., Xie A. (2019). Prion-Like Mechanisms in Parkinson’s Disease. Front. Neurosci..

[14] Baba M., Nakajo S., Tu P.H., Tomita T., Nakaya K., Lee V.M., Trojanowski J.Q., Iwatsubo T. (1998). Aggregation of Alpha-Synuclein in Lewy Bodies of Sporadic Parkinson’s Disease and Dementia with Lewy Bodies. Am. J. Pathol..

[15] Cookson M.R. (2012). Parkinsonism Due to Mutations in PINK1, Parkin, and DJ-1 and Oxidative Stress and Mitochondrial Pathways. Cold Spring Harb. Perspect. Med..

[16] Nuytemans K., Theuns J., Cruts M., Van Broeckhoven C. (2010). Genetic Etiology of Parkinson Disease Associated with Mutations in the SNCA, PARK2, PINK1, PARK7, and LRRK2 Genes: A Mutation Update. Hum. Mutat..

[17] Braak H., Rüb U., Gai W.P., Del Tredici K. (2003). Idiopathic Parkinson’s Disease: Possible Routes by Which Vulnerable Neuronal Types May Be Subject to Neuroinvasion by an Unknown Pathogen. J. Neural Transm..

[18] Müller A., Reichmann H., Livermore A., Hummel T. (2002). Olfactory Function in Idiopathic Parkinson’s Disease (IPD): Results from Cross-Sectional Studies in IPD Patients and Long-Term Follow-up of de-Novo IPD Patients. J. Neural Transm..

[19] Haehner A., Boesveldt S., Berendse H.W., Mackay-Sim A., Fleischmann J., Silburn P.A., Johnston A.N., Mellick G.D., Herting B., Reichmann H. (2009). Prevalence of Smell Loss in Parkinson’s Disease—A Multicenter Study. Parkinsonism Relat. Disord..

[20] Huang H., Xu H., Luo Q., He J., Li M., Chen H., Tang W., Nie Y., Zhou Y. (2019). Fecal Microbiota Transplantation to Treat Parkinson’s Disease with Constipation: A Case Report. Medicine.

[21] Frazzitta G., Ferrazzoli D., Folini A., Palamara G., Maestri R. (2019). Severe Constipation in Parkinson’s Disease and in Parkinsonisms: Prevalence and Affecting Factors. Front. Neurol..

[22] Yu Q.-J., Yu S.-Y., Zuo L.-J., Lian T.-H., Hu Y., Wang R.-D., Piao Y.-S., Guo P., Liu L., Jin Z. (2018). Parkinson Disease with Constipation: Clinical Features and Relevant Factors. Sci. Rep..

[23] Minguez-Castellanos A., Chamorro C.E., Escamilla-Sevilla F., Ortega-Moreno A., Rebollo A.C., Gomez-Rio M., Concha A., Munoz D.G. (2007). Do Alpha-Synuclein Aggregates in Autonomic Plexuses Predate Lewy Body Disorders?: A Cohort Study. Neurology.

[24] Yan F., Chen Y., Li M., Wang Y., Zhang W., Chen X., Ye Q. (2018). Gastrointestinal Nervous System α-Synuclein as a Potential Biomarker of Parkinson Disease. Medicine.

[25] Chandra R., Hiniker A., Kuo Y.-M., Nussbaum R.L., Liddle R.A. (2017). α-Synuclein in Gut Endocrine Cells and Its Implications for Parkinson’s Disease. JCI Insight.

[26] Sampson T.R., Debelius J.W., Thron T., Janssen S., Shastri G.G., Ilhan Z.E., Challis C., Schretter C.E., Rocha S., Gradinaru V. (2016). Gut Microbiota Regulate Motor Deficits and Neuroinflammation in a Model of Parkinson’s Disease. Cell.

[27] Park B.S., Lee J.-O. (2013). Recognition of Lipopolysaccharide Pattern by TLR4 Complexes. Exp. Mol. Med..

[28] Wang J.H., Manning B.J., Wu Q.D., Blankson S., Bouchier-Hayes D., Redmond H.P. (2003). Endotoxin/Lipopolysaccharide Activates NF-Kappa B and Enhances Tumor Cell Adhesion and Invasion through a Beta 1 Integrin-Dependent Mechanism. J. Immunol..

[29] Pajares M., Rojo A.I., Manda G., Boscá L., Cuadrado A. (2020). Inflammation in Parkinson’s Disease: Mechanisms and Therapeutic Implications. Cells.

[30] Pober J.S., Sessa W.C. (2014). Inflammation and the Blood Microvascular System. Cold Spring Harb. Perspect. Biol..

[31] Püntener U., Booth S.G., Perry V.H., Teeling J.L. (2012). Long-Term Impact of Systemic Bacterial Infection on the Cerebral Vasculature and Microglia. J. Neuroinflamm..

[32] Kim C., Lv G., Lee J.S., Jung B.C., Masuda-Suzukake M., Hong C.-S., Valera E., Lee H.-J., Paik S.R., Hasegawa M. (2016). Exposure to Bacterial Endotoxin Generates a Distinct Strain of α-Synuclein Fibril. Sci. Rep..

[33] Hyvärinen T., Hagman S., Ristola M., Sukki L., Veijula K., Kreutzer J., Kallio P., Narkilahti S. (2019). Co-Stimulation with IL-1β and TNF-α Induces an Inflammatory Reactive Astrocyte Phenotype with Neurosupportive Characteristics in a Human Pluripotent Stem Cell Model System. Sci. Rep..

[34] Wang B., Su C.-J., Liu T.-T., Zhou Y., Feng Y., Huang Y., Liu X., Wang Z.-H., Chen L.-H., Luo W.-F. (2018). The Neuroprotection of Low-Dose Morphine in Cellular and Animal Models of Parkinson’s Disease Through Ameliorating Endoplasmic Reticulum (ER) Stress and Activating Autophagy. Front. Mol. Neurosci..

[35] Racette B.A., Gross A., Vouri S.M., Camacho-Soto A., Willis A.W., Searles Nielsen S. (2018). Immunosuppressants and Risk of Parkinson Disease. Ann. Clin. Transl. Neurol..

[36] Brochard V., Combadière B., Prigent A., Laouar Y., Perrin A., Beray-Berthat V., Bonduelle O., Alvarez-Fischer D., Callebert J., Launay J.-M. (2009). Infiltration of CD4^+^ Lymphocytes into the Brain Contributes to Neurodegeneration in a Mouse Model of Parkinson Disease. J. Clin. Invest..

[37] Codolo G., Plotegher N., Pozzobon T., Brucale M., Tessari I., Bubacco L., de Bernard M. (2013). Triggering of Inflammasome by Aggregated α-Synuclein, an Inflammatory Response in Synucleinopathies. PLoS ONE.

[38] Challis C., Hori A., Sampson T.R., Yoo B.B., Challis R.C., Hamilton A.M., Mazmanian S.K., Volpicelli-Daley L.A., Gradinaru V. (2020). Gut-Seeded α-Synuclein Fibrils Promote Gut Dysfunction and Brain Pathology Specifically in Aged Mice. Nat. Neurosci..

[39] Kim S., Kwon S.-H., Kam T.-I., Panicker N., Karuppagounder S.S., Lee S., Lee J.H., Kim W.R., Kook M., Foss C.A. (2019). Transneuronal Propagation of Pathologic α-Synuclein from the Gut to the Brain Models Parkinson’s Disease. Neuron.

[40] Milan Manani S., Virzì G.M., Giuliani A., Baretta M., Corradi V., De Cal M., Biasi C., Crepaldi C., Ronco C. (2020). Lipopolysaccharide Evaluation in Peritoneal Dialysis Patients with Peritonitis. Blood Purif..

[41] Petrov V.A., Saltykova I.V., Zhukova I.A., Alifirova V.M., Zhukova N.G., Dorofeeva Y.B., Tyakht A.V., Kovarsky B.A., Alekseev D.G., Kostryukova E.S. (2017). Analysis of Gut Microbiota in Patients with Parkinson’s Disease. Bull. Exp. Biol. Med..

[42] Scheperjans F., Aho V., Pereira P.A.B., Koskinen K., Paulin L., Pekkonen E., Haapaniemi E., Kaakkola S., Eerola-Rautio J., Pohja M. (2015). Gut Microbiota Are Related to Parkinson’s Disease and Clinical Phenotype. Mov. Disord..

[43] Forsyth C.B., Shannon K.M., Kordower J.H., Voigt R.M., Shaikh M., Jaglin J.A., Estes J.D., Dodiya H.B., Keshavarzian A. (2011). Increased Intestinal Permeability Correlates with Sigmoid Mucosa Alpha-Synuclein Staining and Endotoxin Exposure Markers in Early Parkinson’s Disease. PLoS ONE.

[44] Sampson T.R., Challis C., Jain N., Moiseyenko A., Ladinsky M.S., Shastri G.G., Thron T., Needham B.D., Horvath I., Debelius J.W. (2020). A Gut Bacterial Amyloid Promotes α-Synuclein Aggregation and Motor Impairment in Mice. eLife.

[45] Romano S., Savva G.M., Bedarf J.R., Charles I.G., Hildebrand F., Narbad A. (2021). Meta-Analysis of the Parkinson’s Disease Gut Microbiome Suggests Alterations Linked to Intestinal Inflammation. NPJ Park. Dis..

[46] Alhadhrami G., Huber J.T., Higginbotham G.E., Harper J.M. (1989). Nutritive Value of High Moisture Alfalfa Hay Preserved with Urea. J. Dairy Sci..

[47] Zhu L., Liu W., Alkhouri R., Baker R.D., Bard J.E., Quigley E.M., Baker S.S. (2014). Structural Changes in the Gut Microbiome of Constipated Patients. Physiol. Genom..

[48] Esquivel-Elizondo S., Ilhan Z.E., Garcia-Peña E.I., Krajmalnik-Brown R. (2017). Insights into Butyrate Production in a Controlled Fermentation System via Gene Predictions. mSystems.

[49] Haikal C., Chen Q.-Q., Li J.-Y. (2019). Microbiome Changes: An Indicator of Parkinson’s Disease?. Transl. Neurodegener..

[50] Segain J.-P. (2000). Butyrate Inhibits Inflammatory Responses through NFkappa B Inhibition: Implications for Crohn’s Disease. Gut.

[51] Liu J., Wang F., Liu S., Du J., Hu X., Xiong J., Fang R., Chen W., Sun J. (2017). Sodium Butyrate Exerts Protective Effect against Parkinson’s Disease in Mice via Stimulation of Glucagon like Peptide-1. J. Neurol. Sci..

[52] Qiao C.-M., Sun M.-F., Jia X.-B., Li Y., Zhang B.-P., Zhao L.-P., Shi Y., Zhou Z.-L., Zhu Y.-L., Cui C. (2020). Sodium Butyrate Exacerbates Parkinson’s Disease by Aggravating Neuroinflammation and Colonic Inflammation in MPTP-Induced Mice Model. Neurochem. Res..

[53] Warren J.R., Marshall B. (1983). Unidentified Curved Bacilli on Gastric Epithelium in Active Chronic Gastritis. Lancet.

[54] Polk D.B., Peek R.M. (2010). Helicobacter Pylori: Gastric Cancer and Beyond. Nat. Rev. Cancer.

[55] Çamcı G., Oğuz S. (2016). Association between Parkinson’s Disease and Helicobacter Pylori. J. Clin. Neurol..

[56] Takahashi M., Yamada T. (1999). Viral Etiology for Parkinson’s Disease—A Possible Role of Influenza A Virus Infection. Jpn. J. Infect. Dis..

[57] Jang H., Boltz D.A., Webster R.G., Smeyne R.J. (2009). Viral Parkinsonism. Biochim. Biophys. Acta.

[58] Hoffman L.A., Vilensky J.A. (2017). Encephalitis Lethargica: 100 Years after the Epidemic. Brain.

[59] Maurizi C.P. (2010). Influenza Caused Epidemic Encephalitis (Encephalitis Lethargica): The Circumstantial Evidence and a Challenge to the Nonbelievers. Med. Hypotheses.

[60] Valero-Pacheco N., Arriaga-Pizano L., Ferat-Osorio E., Mora-Velandia L.M., Pastelin-Palacios R., Villasís-Keever M.Á., Alpuche-Aranda C., Sánchez-Torres L.E., Isibasi A., Bonifaz L. (2013). PD-L1 Expression Induced by the 2009 Pandemic Influenza A(H1N1) Virus Impairs the Human T Cell Response. Clin. Dev. Immunol..

[61] Osborne O., Peyravian N., Nair M., Daunert S., Toborek M. (2020). The Paradox of HIV Blood–Brain Barrier Penetrance and Antiretroviral Drug Delivery Deficiencies. Trends Neurosci..

[62] Ren R., Racaniello V.R. (1992). Poliovirus Spreads from Muscle to the Central Nervous System by Neural Pathways. J. Infect. Dis..

[63] Young V.A., Rall G.F. (2009). Making It to the Synapse: Measles Virus Spread in and among Neurons. Curr. Top. Microbiol. Immunol..

[64] Sulzer D., Antonini A., Leta V., Nordvig A., Smeyne R.J., Goldman J.E., Al-Dalahmah O., Zecca L., Sette A., Bubacco L. (2020). COVID-19 and Possible Links with Parkinson’s Disease and Parkinsonism: From Bench to Bedside. NPJ Parkinsons Dis..

[65] Loewy A.D. (1998). Viruses as Transneuronal Tracers for Defining Neural Circuits. Neurosci. Biobehav. Rev..

[66] Bedarf J.R., Hildebrand F., Coelho L.P., Sunagawa S., Bahram M., Goeser F., Bork P., Wüllner U. (2017). Functional Implications of Microbial and Viral Gut Metagenome Changes in Early Stage L-DOPA-Naïve Parkinson’s Disease Patients. Genome Med..

[67] Mertsalmi T.H., Pekkonen E., Scheperjans F. (2020). Antibiotic Exposure and Risk of Parkinson’s Disease in Finland: A Nationwide Case-Control Study. Mov. Disord. Off. J. Mov. Disord. Soc..

[68] Cleophas M.C.P., Ratter J.M., Bekkering S., Quintin J., Schraa K., Stroes E.S., Netea M.G., Joosten L.A.B. (2019). Effects of Oral Butyrate Supplementation on Inflammatory Potential of Circulating Peripheral Blood Mononuclear Cells in Healthy and Obese Males. Sci. Rep..

[69] Van Kessel S.P., Frye A.K., El-Gendy A.O., Castejon M., Keshavarzian A., van Dijk G., El Aidy S. (2019). Gut Bacterial Tyrosine Decarboxylases Restrict Levels of Levodopa in the Treatment of Parkinson’s Disease. Nat. Commun..

[70] Bae H.-G., Kim T.K., Suk H.Y., Jung S., Jo D.-G. (2020). White Matter and Neurological Disorders. Arch. Pharm. Res..

[71] Van Cauwenberghe C., Van Broeckhoven C., Sleegers K. (2016). The Genetic Landscape of Alzheimer Disease: Clinical Implications and Perspectives. Genet. Med..

[72] Attems J., Jellinger K.A. (2014). The Overlap between Vascular Disease and Alzheimer’s Disease—Lessons from Pathology. BMC Med..

[73] Kapasi A., Schneider J.A. (2016). Vascular Contributions to Cognitive Impairment, Clinical Alzheimer’s Disease, and Dementia in Older Persons. Biochim. Biophys. Acta BBA—Mol. Basis Dis..

[74] Selkoe D.J., Hardy J. (2016). The Amyloid Hypothesis of Alzheimer’s Disease at 25 Years. EMBO Mol. Med..

[75] Drews A., Flint J., Shivji N., Jönsson P., Wirthensohn D., De Genst E., Vincke C., Muyldermans S., Dobson C., Klenerman D. (2016). Individual Aggregates of Amyloid Beta Induce Temporary Calcium Influx through the Cell Membrane of Neuronal Cells. Sci. Rep..

[76] Ding X., Zhang M., Gu R., Xu G., Wu H. (2017). Activated Microglia Induce the Production of Reactive Oxygen Species and Promote Apoptosis of Co-Cultured Retinal Microvascular Pericytes. Graefes Arch. Clin. Exp. Ophthalmol..

[77] Lull M.E., Block M.L. (2010). Microglial Activation and Chronic Neurodegeneration. Neurotherapeutics.

[78] Cuchillo-Ibanez I., Seereeram A., Byers H.L., Leung K., Ward M.A., Anderton B.H., Hanger D.P. (2008). Phosphorylation of Tau Regulates Its Axonal Transport by Controlling Its Binding to Kinesin. FASEB J..

[79] Combs B., Mueller R.L., Morfini G., Brady S.T., Kanaan N.M. (2019). Tau and Axonal Transport Misregulation in Tauopathies. Adv. Exp. Med. Biol..

[80] Aducanumab Still Needs to Prove Itself, Researchers Say/ALZFORUM. https://www.alzforum.org/news/research-news/aducanumab-still-needs-prove-itself-researchers-say.

[81] Honig L.S., Vellas B., Woodward M., Boada M., Bullock R., Borrie M., Hager K., Andreasen N., Scarpini E., Liu-Seifert H. (2018). Trial of Solanezumab for Mild Dementia Due to Alzheimer’s Disease. N. Engl. J. Med..

[82] Mehta D., Jackson R., Paul G., Shi J., Sabbagh M. (2017). Why Do Trials for Alzheimer’s Disease Drugs Keep Failing? A Discontinued Drug Perspective for 2010–2015. Expert Opin. Investig. Drugs.

[83] Congdon E.E., Sigurdsson E.M. (2018). Tau-Targeting Therapies for Alzheimer Disease. Nat. Rev. Neurol..

[84] Killin L.O.J., Starr J.M., Shiue I.J., Russ T.C. (2016). Environmental Risk Factors for Dementia: A Systematic Review. BMC Geriatr..

[85] Griciuc A., Patel S., Federico A.N., Choi S.H., Innes B.J., Oram M.K., Cereghetti G., McGinty D., Anselmo A., Sadreyev R.I. (2019). TREM2 Acts Downstream of CD33 in Modulating Microglial Pathology in Alzheimer’s Disease. Neuron.

[86] Zhuang Z.-Q., Shen L.-L., Li W.-W., Fu X., Zeng F., Gui L., Lü Y., Cai M., Zhu C., Tan Y.-L. (2018). Gut Microbiota Is Altered in Patients with Alzheimer’s Disease. J. Alzheimers Dis. JAD.

[87] Haran J.P., Bhattarai S.K., Foley S.E., Dutta P., Ward D.V., Bucci V., McCormick B.A. (2019). Alzheimer’s Disease Microbiome Is Associated with Dysregulation of the Anti-Inflammatory P-Glycoprotein Pathway. mBio.

[88] Chapman M.R. (2002). Role of *Escherichia coli* Curli Operons in Directing Amyloid Fiber Formation. Science.

[89] Cherny I., Rockah L., Levy-Nissenbaum O., Gophna U., Ron E.Z., Gazit E. (2005). The Formation of Escherichia Coli Curli Amyloid Fibrils Is Mediated by Prion-like Peptide Repeats. J. Mol. Biol..

[90] Reichhardt C., Lim J.Y., Rice D., Fong J.N., Cegelski L. (2014). Structure and Function of Bacterial Biofilms by Solid-State NMR. Biophys. J..

[91] Lundmark K., Westermark G.T., Olsen A., Westermark P. (2005). Protein Fibrils in Nature Can Enhance Amyloid Protein a Amyloidosis in Mice: Cross-Seeding as a Disease Mechanism. Proc. Natl. Acad. Sci. USA.

[92] Chen S.G., Stribinskis V., Rane M.J., Demuth D.R., Gozal E., Roberts A.M., Jagadapillai R., Liu R., Choe K., Shivakumar B. (2016). Exposure to the Functional Bacterial Amyloid Protein Curli Enhances Alpha-Synuclein Aggregation in Aged Fischer 344 Rats and Caenorhabditis Elegans. Sci. Rep..

[93] Friedland R.P. (2015). Mechanisms of Molecular Mimicry Involving the Microbiota in Neurodegeneration. J. Alzheimers Dis..

[94] Friedland R.P., Chapman M.R. (2017). The Role of Microbial Amyloid in Neurodegeneration. PLoS Pathog..

[95] Mezö C., Dokalis N., Mossad O., Staszewski O., Neuber J., Yilmaz B., Schnepf D., de Agüero M.G., Ganal-Vonarburg S.C., Macpherson A.J. (2020). Different Effects of Constitutive and Induced Microbiota Modulation on Microglia in a Mouse Model of Alzheimer’s Disease. Acta Neuropathol. Commun..

[96] Fujii Y., Nguyen T.T.T., Fujimura Y., Kameya N., Nakamura S., Arakawa K., Morita H. (2019). Fecal Metabolite of a Gnotobiotic Mouse Transplanted with Gut Microbiota from a Patient with Alzheimer’s Disease. Biosci. Biotechnol. Biochem..

[97] He Y., Li B., Sun D., Chen S. (2020). Gut Microbiota: Implications in Alzheimer’s Disease. J. Clin. Med..

[98] Wang T., Hu X., Liang S., Li W., Wu X., Wang L., Jin F. (2015). Lactobacillus Fermentum NS9 Restores the Antibiotic Induced Physiological and Psychological Abnormalities in Rats. Benef. Microbes.

[99] Agahi A., Hamidi G.A., Daneshvar R., Hamdieh M., Soheili M., Alinaghipour A., Esmaeili Taba S.M., Salami M. (2018). Does Severity of Alzheimer’s Disease Contribute to Its Responsiveness to Modifying Gut Microbiota? A Double Blind Clinical Trial. Front. Neurol..

[100] Leblhuber F., Steiner K., Schuetz B., Fuchs D., Gostner J.M. (2018). Probiotic Supplementation in Patients with Alzheimer’s Dementia—An Explorative Intervention Study. Curr. Alzheimer Res..

[101] Geirnaert A., Calatayud M., Grootaert C., Laukens D., Devriese S., Smagghe G., De Vos M., Boon N., Van de Wiele T. (2017). Butyrate-Producing Bacteria Supplemented in Vitro to Crohn’s Disease Patient Microbiota Increased Butyrate Production and Enhanced Intestinal Epithelial Barrier Integrity. Sci. Rep..

[102] Harach T., Marungruang N., Duthilleul N., Cheatham V., Mc Coy K.D., Frisoni G., Neher J.J., Fåk F., Jucker M., Lasser T. (2017). Reduction of Abeta Amyloid Pathology in APPPS1 Transgenic Mice in the Absence of Gut Microbiota. Sci. Rep..

[103] Cox L.M., Schafer M.J., Sohn J., Vincentini J., Weiner H.L., Ginsberg S.D., Blaser M.J. (2019). Calorie Restriction Slows Age-Related Microbiota Changes in an Alzheimer’s Disease Model in Female Mice. Sci. Rep..

[104] Dodiya H.B., Frith M., Sidebottom A., Cao Y., Koval J., Chang E., Sisodia S.S. (2020). Synergistic Depletion of Gut Microbial Consortia, but Not Individual Antibiotics, Reduces Amyloidosis in APPPS1-21 Alzheimer’s Transgenic Mice. Sci. Rep..

[105] Gubert C., Kong G., Renoir T., Hannan A.J. (2020). Exercise, Diet and Stress as Modulators of Gut Microbiota: Implications for Neurodegenerative Diseases. Neurobiol. Dis..

[106] Desai M.S., Seekatz A.M., Koropatkin N.M., Kamada N., Hickey C.A., Wolter M., Pudlo N.A., Kitamoto S., Terrapon N., Muller A. (2016). A Dietary Fiber-Deprived Gut Microbiota Degrades the Colonic Mucus Barrier and Enhances Pathogen Susceptibility. Cell.

[107] Parada Venegas D., De la Fuente M.K., Landskron G., González M.J., Quera R., Dijkstra G., Harmsen H.J.M., Faber K.N., Hermoso M.A. (2019). Short Chain Fatty Acids (SCFAs)-Mediated Gut Epithelial and Immune Regulation and Its Relevance for Inflammatory Bowel Diseases. Front. Immunol..

[108] Koh A., De Vadder F., Kovatcheva-Datchary P., Bäckhed F. (2016). From Dietary Fiber to Host Physiology: Short-Chain Fatty Acids as Key Bacterial Metabolites. Cell.

[109] Wu G.D., Chen J., Hoffmann C., Bittinger K., Chen Y.-Y., Keilbaugh S.A., Bewtra M., Knights D., Walters W.A., Knight R. (2011). Linking Long-Term Dietary Patterns with Gut Microbial Enterotypes. Science.

[110] Riccio P., Rossano R. (2018). Diet, Gut Microbiota, and Vitamins D + A in Multiple Sclerosis. Neurotherapeutics.

[111] Zhernakova A., Kurilshikov A., Bonder M.J., Tigchelaar E.F., Schirmer M., Vatanen T., Mujagic Z., Vila A.V., Falony G., Vieira-Silva S. (2016). Population-Based Metagenomics Analysis Reveals Markers for Gut Microbiome Composition and Diversity. Science.

[112] Turnbaugh P.J., Bäckhed F., Fulton L., Gordon J.I. (2008). Diet-Induced Obesity Is Linked to Marked but Reversible Alterations in the Mouse Distal Gut Microbiome. Cell Host Microbe.

[113] Zhang H., Tang K., Ma J., Zhou L., Liu J., Zeng L., Zhu L., Xu P., Chen J., Wei K. (2020). Ketogenesis-Generated β-Hydroxybutyrate Is an Epigenetic Regulator of CD8+ T-Cell Memory Development. Nat. Cell Biol..

[114] Barborka C.J. (1928). Ketogenic Diet Treatment of Epilepsy in Adults. J. Am. Med. Assoc..

[115] Maiorana A., Manganozzi L., Barbetti F., Bernabei S., Gallo G., Cusmai R., Caviglia S., Dionisi-Vici C. (2015). Ketogenic Diet in a Patient with Congenital Hyperinsulinism: A Novel Approach to Prevent Brain Damage. Orphanet J. Rare Dis..

[116] Ma D., Wang A.C., Parikh I., Green S.J., Hoffman J.D., Chlipala G., Murphy M.P., Sokola B.S., Bauer B., Hartz A.M.S. (2018). Ketogenic Diet Enhances Neurovascular Function with Altered Gut Microbiome in Young Healthy Mice. Sci. Rep..

[117] Ang Q.Y., Alexander M., Newman J.C., Tian Y., Cai J., Upadhyay V., Turnbaugh J.A., Verdin E., Hall K.D., Leibel R.L. (2020). Ketogenic Diets Alter the Gut Microbiome Resulting in Decreased Intestinal Th17 Cells. Cell.

[118] Wu L., Sun D. (2017). Adherence to Mediterranean Diet and Risk of Developing Cognitive Disorders: An Updated Systematic Review and Meta-Analysis of Prospective Cohort Studies. Sci. Rep..

[119] Wang D.D., Nguyen L.H., Li Y., Yan Y., Ma W., Rinott E., Ivey K.L., Shai I., Willett W.C., Hu F.B. (2021). The Gut Microbiome Modulates the Protective Association between a Mediterranean Diet and Cardiometabolic Disease Risk. Nat. Med..

[120] Hoffman J.D., Yanckello L.M., Chlipala G., Hammond T.C., McCulloch S.D., Parikh I., Sun S., Morganti J.M., Green S.J., Lin A.-L. (2019). Dietary Inulin Alters the Gut Microbiome, Enhances Systemic Metabolism and Reduces Neuroinflammation in an APOE4 Mouse Model. PLoS ONE.

[121] Millman J.F., Okamoto S., Teruya T., Uema T., Ikematsu S., Shimabukuro M., Masuzaki H. (2021). Extra-Virgin Olive Oil and the Gut-Brain Axis: Influence on Gut Microbiota, Mucosal Immunity, and Cardiometabolic and Cognitive Health. Nutr. Rev..

[122] Morris M.C., Evans D.A., Bienias J.L., Tangney C.C., Bennett D.A., Aggarwal N., Schneider J., Wilson R.S. (2003). Dietary Fats and the Risk of Incident Alzheimer Disease. Arch. Neurol..

[123] Fu Y., Wang Y., Gao H., Li D., Jiang R., Ge L., Tong C., Xu K. (2021). Associations among Dietary Omega-3 Polyunsaturated Fatty Acids, the Gut Microbiota, and Intestinal Immunity. Mediat. Inflamm..

[124] McCarty M.F. (2001). Does a Vegan Diet Reduce Risk for Parkinson’s Disease?. Med. Hypotheses.

[125] So D., Whelan K., Rossi M., Morrison M., Holtmann G., Kelly J.T., Shanahan E.R., Staudacher H.M., Campbell K.L. (2018). Dietary Fiber Intervention on Gut Microbiota Composition in Healthy Adults: A Systematic Review and Meta-Analysis. Am. J. Clin. Nutr..

[126] Million M., Tidjani-Alou M., Khelaifia S., Bachar D., Lagier J.-C., Dione N., Brah S., Hugon P., Lombard V., Armougom F. (2016). Increased Gut Redox and Depletion of Anaerobic and Methanogenic Prokaryotes in Severe Acute Malnutrition. Sci. Rep..

[127] Million M., Armstrong N., Khelaifia S., Guilhot E., Richez M., Lagier J.-C., Dubourg G., Chabriere E., Raoult D. (2020). The Antioxidants Glutathione, Ascorbic Acid and Uric Acid Maintain Butyrate Production by Human Gut Clostridia in The Presence of Oxygen In Vitro. Sci. Rep..

[128] Liu H.-X., Rocha C.S., Dandekar S., Wan Y.-J.Y. (2016). Functional Analysis of the Relationship between Intestinal Microbiota and the Expression of Hepatic Genes and Pathways during the Course of Liver Regeneration. J. Hepatol..

[129] Hegelmaier T., Lebbing M., Duscha A., Tomaske L., Tönges L., Holm J.B., Bjørn Nielsen H., Gatermann S.G., Przuntek H., Haghikia A. (2020). Interventional Influence of the Intestinal Microbiome Through Dietary Intervention and Bowel Cleansing Might Improve Motor Symptoms in Parkinson’s Disease. Cells.

[130] Clavel T., Fallani M., Lepage P., Levenez F., Mathey J., Rochet V., Sérézat M., Sutren M., Henderson G., Bennetau-Pelissero C. (2005). Isoflavones and Functional Foods Alter the Dominant Intestinal Microbiota in Postmenopausal Women. J. Nutr..

[131] Gao X., Xie Q., Kong P., Liu L., Sun S., Xiong B., Huang B., Yan L., Sheng J., Xiang H. (2017). Polyphenol- and Caffeine-Rich Postfermented Pu-Erh Tea Improves Diet-Induced Metabolic Syndrome by Remodeling Intestinal Homeostasis in Mice. Infect. Immun..

[132] Tzounis X., Rodriguez-Mateos A., Vulevic J., Gibson G.R., Kwik-Uribe C., Spencer J.P. (2011). Prebiotic Evaluation of Cocoa-Derived Flavanols in Healthy Humans by Using a Randomized, Controlled, Double-Blind, Crossover Intervention Study. Am. J. Clin. Nutr..

[133] Ma G., Chen Y. (2020). Polyphenol Supplementation Benefits Human Health via Gut Microbiota: A Systematic Review via Meta-Analysis. J. Funct. Foods.

[134] Hidalgo-Liberona N., González-Domínguez R., Vegas E., Riso P., Del Bo’ C., Bernardi S., Peron G., Guglielmetti S., Gargari G., Kroon P.A. (2020). Increased Intestinal Permeability in Older Subjects Impacts the Beneficial Effects of Dietary Polyphenols by Modulating Their Bioavailability. J. Agric. Food Chem..

[135] Koliaki C., Spinos T., Spinou Μ., Brinia Μ.-E., Mitsopoulou D., Katsilambros N. (2018). Defining the Optimal Dietary Approach for Safe, Effective and Sustainable Weight Loss in Overweight and Obese Adults. Healthcare.

[136] Harris L., Hamilton S., Azevedo L.B., Olajide J., De Brún C., Waller G., Whittaker V., Sharp T., Lean M., Hankey C. (2018). Intermittent Fasting Interventions for Treatment of Overweight and Obesity in Adults: A Systematic Review and Meta-Analysis. JBI Database Syst. Rev. Implement. Rep..

[137] Zhang C., Li S., Yang L., Huang P., Li W., Wang S., Zhao G., Zhang M., Pang X., Yan Z. (2013). Structural Modulation of Gut Microbiota in Life-Long Calorie-Restricted Mice. Nat. Commun..

[138] Behl C., Schubert D. (1993). Heat Shock Partially Protects Rat Pheochromocytoma PC12 Cells from Amyloid β Peptide Toxicity. Neurosci. Lett..

[139] Ehrenfried J.A., Evers B.M., Chu K.U., Townsend C.M., Thompson J.C. (1996). Caloric Restriction Increases the Expression of Heat Shock Protein in the Gut. Ann. Surg..

[140] Graff J., Kahn M., Samiei A., Gao J., Ota K.T., Rei D., Tsai L.-H. (2013). A Dietary Regimen of Caloric Restriction or Pharmacological Activation of SIRT1 to Delay the Onset of Neurodegeneration. J. Neurosci..

[141] Zou H., Wang D., Ren H., Cai K., Chen P., Fang C., Shi Z., Zhang P., Wang J., Yang H. (2020). Effect of Caloric Restriction on BMI, Gut Microbiota, and Blood Amino Acid Levels in Non-Obese Adults. Nutrients.

[142] Pifferi F., Terrien J., Marchal J., Dal-Pan A., Djelti F., Hardy I., Chahory S., Cordonnier N., Desquilbet L., Hurion M. (2018). Caloric Restriction Increases Lifespan but Affects Brain Integrity in Grey Mouse Lemur Primates. Commun. Biol..

[143] Redman L.M., Ravussin E. (2011). Caloric Restriction in Humans: Impact on Physiological, Psychological, and Behavioral Outcomes. Antioxid. Redox Signal..

[144] Tamtaji O.R., Taghizadeh M., Daneshvar Kakhaki R., Kouchaki E., Bahmani F., Borzabadi S., Oryan S., Mafi A., Asemi Z. (2019). Clinical and Metabolic Response to Probiotic Administration in People with Parkinson’s Disease: A Randomized, Double-Blind, Placebo-Controlled Trial. Clin. Nutr..

[145] Georgescu D., Ancusa O., Georgescu L., Ionita I., Reisz D. (2016). Nonmotor Gastrointestinal Disorders in Older Patients with Parkinson&rsquos Disease: Is There Hope?. Clin. Interv. Aging.

[146] Barichella M., Pacchetti C., Bolliri C., Cassani E., Iorio L., Pusani C., Pinelli G., Privitera G., Cesari I., Faierman S.A. (2016). Probiotics and Prebiotic Fiber for Constipation Associated with Parkinson Disease: An RCT. Neurology.

[147] Cassani E., Privitera G., Pezzoli G., Pusani C., Madio C., Iorio L., Barichella M. (2011). Use of Probiotics for the Treatment of Constipation in Parkinson’s Disease Patients. Minerva Gastroenterol. Dietol..

[148] Lugtenberg B. (1981). Composition and Function of the Outer Membrane of Escherichia Coli. Trends Biochem. Sci..

[149] Kaper J.B., Nataro J.P., Mobley H.L.T. (2004). Pathogenic Escherichia Coli. Nat. Rev. Microbiol..

[150] Llorente B., de Souza F.S.J., Soto G., Meyer C., Alonso G.D., Flawiá M.M., Bravo-Almonacid F., Ayub N.D., Rodríguez-Concepción M. (2016). Selective Pressure against Horizontally Acquired Prokaryotic Genes as a Driving Force of Plastid Evolution. Sci. Rep..

[151] Abraham D., Feher J., Scuderi G.L., Szabo D., Dobolyi A., Cservenak M., Juhasz J., Ligeti B., Pongor S., Gomez-Cabrera M.C. (2019). Exercise and Probiotics Attenuate the Development of Alzheimer’s Disease in Transgenic Mice: Role of Microbiome. Exp. Gerontol..

[152] Sonnenburg E.D., Smits S.A., Tikhonov M., Higginbottom S.K., Wingreen N.S., Sonnenburg J.L. (2016). Diet-Induced Extinctions in the Gut Microbiota Compound over Generations. Nature.

[153] De la Rosa A., Solana E., Corpas R., Bartrés-Faz D., Pallàs M., Vina J., Sanfeliu C., Gomez-Cabrera M.C. (2019). Long-Term Exercise Training Improves Memory in Middle-Aged Men and Modulates Peripheral Levels of BDNF and Cathepsin B. Sci. Rep..

[154] Sleiman S.F., Henry J., Al-Haddad R., El Hayek L., Abou Haidar E., Stringer T., Ulja D., Karuppagounder S.S., Holson E.B., Ratan R.R. (2016). Exercise Promotes the Expression of Brain Derived Neurotrophic Factor (BDNF) through the Action of the Ketone Body β-Hydroxybutyrate. eLife.

[155] Menon R., Fitzsimmons B., Vanajakumari M.U., Lee K., Jayaraman A. (2019). Effect of Norepinephrine on Gut Bacterial Community Structure and Function. Faseb J..

[156] Antoni M.H., Cruess D.G., Cruess S., Lutgendorf S., Kumar M., Ironson G., Klimas N., Fletcher M.A., Schneiderman N. (2000). Cognitive–Behavioral Stress Management Intervention Effects on Anxiety, 24-Hr Urinary Norepinephrine Output, and T-Cytotoxic/Suppressor Cells over Time among Symptomatic HIV-Infected Gay Men. J. Consult. Clin. Psychol..

[157] Mudd A.T., Berding K., Wang M., Donovan S.M., Dilger R.N. (2017). Serum Cortisol Mediates the Relationship between Fecal *Ruminococcus* and Brain N-Acetylaspartate in the Young Pig. Gut Microbes.

[158] Abelson J.L., Liberzon I., Young E.A., Khan S. (2005). Cognitive Modulation of the Endocrine Stress Response to a Pharmacological Challenge in Normal and Panic Disorder Subjects. Arch. Gen. Psychiatry.

